# Regeneration in Reptiles Generally and the New Zealand Tuatara in Particular as a Model to Analyse Organ Regrowth in Amniotes: A Review

**DOI:** 10.3390/jdb9030036

**Published:** 2021-08-30

**Authors:** Lorenzo Alibardi, Victor Benno Meyer-Rochow

**Affiliations:** 1 Comparative Histolab Padova and Department of Biology, University of Bologna, 40121 Bologna, Italy; lorenzo.alibardi@unibo.it; 2 Agricultural Science and Technology Research Institute, Andong National University, Andong 36729, Korea; 3 Department of Ecology and Genetics, Oulu University, SF-90140 Oulu, Finland

**Keywords:** reptilia, rhynchocephalia, Squamata, lepidosauria, *Sphenodon*, tail, autotomy, morphogenesis, microscopy

## Abstract

The ability to repair injuries among reptiles, i.e., ectothermic amniotes, is similar to that of mammals with some noteworthy exceptions. While large wounds in turtles and crocodilians are repaired through scarring, the reparative capacity involving the tail derives from a combined process of wound healing and somatic growth, the latter being continuous in reptiles. When the tail is injured in juvenile crocodilians, turtles and tortoises as well as the tuatara (Rhynchocephalia: *Sphenodon punctatus*, Gray 1842), the wound is repaired in these reptiles and some muscle and connective tissue and large amounts of cartilage are regenerated during normal growth. This process, here indicated as “regengrow”, can take years to produce tails with similar lengths of the originals and results in only apparently regenerated replacements. These new tails contain a cartilaginous axis and very small (turtle and crocodilians) to substantial (e.g., in tuatara) muscle mass, while most of the tail is formed by an irregular dense connective tissue containing numerous fat cells and sparse nerves. Tail regengrow in the tuatara is a long process that initially resembles that of lizards (the latter being part of the sister group Squamata within the Lepidosauria) with the formation of an axial ependymal tube isolated within a cartilaginous cylinder and surrounded by an irregular fat-rich connective tissue, some muscle bundles, and neogenic scales. Cell proliferation is active in the apical regenerative blastema, but much reduced cell proliferation continues in older regenerated tails, where it occurs mostly in the axial cartilage and scale epidermis of the new tail, but less commonly in the regenerated spinal cord, muscles, and connective tissues. The higher tissue regeneration of *Sphenodon* and other lepidosaurians provides useful information for attempts to improve organ regeneration in endothermic amniotes.

## 1. Introduction and Overview

### 1.1. Wound Healing and Regeneration among Reptiles Generally

**Figure 1:**
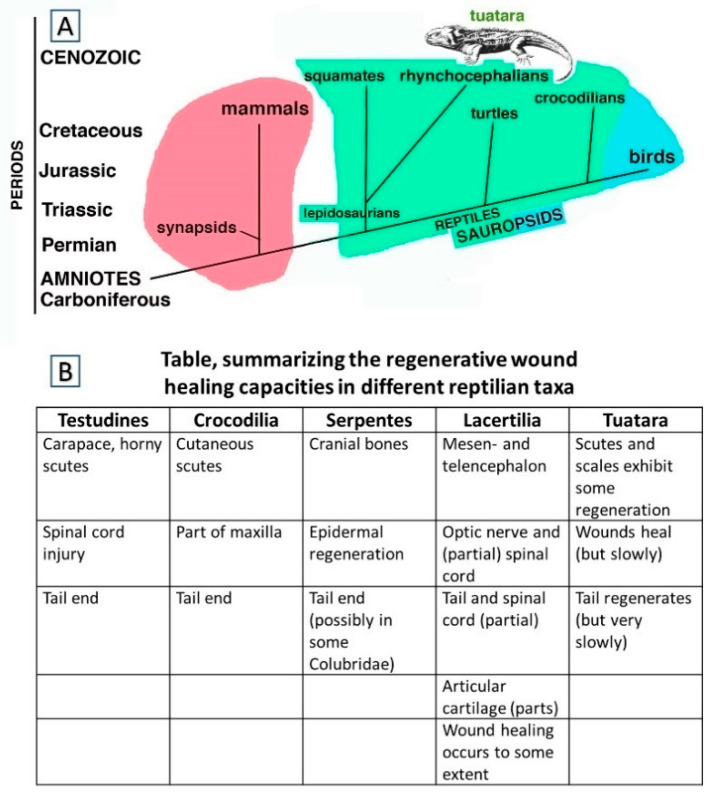
(**A**), Schematic cladogramme indicating the two main amniote radiation paths from basal amniotes, i.e., synapsids-mammals (pink) and sauropsids that include reptiles (green) and birds (light blue). (**B**), Table indicating the main organ and tissues undergoing wound healing with scarring, regengrow and heteromorphic regeneration in different sub-orders of Reptiles.

Extant reptiles represent the modern form of the first amniotes that evolved in the Upper Carboniferous-Lower Permian [1,2]. It has been hypothesized that when amniotes evolved a form of direct development, eliminating the larval growth period and metamorphic phases of their amphibian ancestors, they also lost a number of genes implicated in regeneration [3]. The evolution of reptiles from a common ancestor of “basal amniotes” known as cotylosaurs, resulted in amniotes with a direct embryonic development and an ectothermic metabolism, initiated in the Upper Carboniferous and the Permian. One lineage led to synapsids, with another leading to sauropsids, having already been established (Figure 1A). Among the sauropsids, independent evolutionary lineages separated lepidosaurians from testudines and crocodilians already from the Permian/lower Triassic, and archosaurians eventually gave rise to birds in the Jurassic.

Within the lepidosaurians, the widely used term for the lineage that includes the tuatara and the sister-group of squamates is “Rhynchocephalia” whereas Sphenodontidae refers to just one family within this lineage. Squamates, i.e., scaled reptiles, comprising lizards, snakes, and amphisbaenians, separated during the Triassic and gave rise to reptiles whose living representatives often retain a lizard-like body form [4]. Different from pisciform vertebrates, where wounds or injuries are constantly bathed and flushed by water, in the terrestrial environment small injuries and larger wounds—as well as significant losses of skin, tails or limbs—had to be repaired quickly to avoid water loss and microbe infections. Scarring, a rapid process of healing, was and still is the common outcome in terrestrial vertebrates [5], a process likely to date back to all Mesozoic sauropsids and therapsid-early mammals, especially in cases that involved losses of large organs such as the tail or limbs.

Although mentioned as early as 1886 by Gadow [6], regenerative capabilities in testudines and crocodilians are limited, as indicated in Figure 1B. Injuries in tortoises affect primarily the carapace, the legs, or the tail and can be the result of attacks by large predators or rats, of encounters with machinery such as cars, lawn mowers, etc. and of exposure to fires, but as observations on *Terrapene carolina* by Howey & Roosenburg [7] and on *Trachemys scripta elegans* by Negrini et al. [8] show, wound healing in chelonians is a very slow process. Freshwater and marine turtles are increasingly at risk of being damaged by ships and motor boats. Wound healing and repairs to damaged horny carapaces in tortoises demonstrate a certain regenerative capacity [9,10,11,12] and that some ability exists to at least partially replace a lost or damaged tail has been reported by Davenport [13] for *Testudo hermanni* and Kuchling [14] for *Emydura* sp.

Not a great deal of information is available on the duration of the process in chelonians (Figure 1B), but in *Testudo hermanni* it took the tail spur six years to regenerate to a length of 4 mm and required 12 years to reach 8 mm. At that time, it had regained the pattern and colouration typical of the species. A partial recovery over six years of fire-induced damage to the carapace of a *Testudo hermanni* has been reported by Martinez-Silvestre & Soler-Massana [15]. A bifid tail, described by Mota-Rodrigues & Feitosa-Silva [16] was present in the river turtle *Phrynops tuberosus* and that the freshwater turtle *Trachemys dorbignyi* even recovered from spinal cord injury and regained the competence to use its legs and to walk again, although not as well as before the injury, was demonstrated by Reherman et al. [17,18].

Repairs to part of the mandible and cutaneous scutes in crocodilians have been mentioned, Brazaitis [19] (Figure 1B), but information on regenerative processes involving tails is not extensive, although it has been documented as early as 1937 [20]. Rashid & Chapman [21] have discussed why it is not always easy to precisely define the tail and denote the boundary between it and the trunk and have pointed out that “outgrowth of the tail from the secondary body shares numerous developmental features with limbs”. However, limb regeneration appears to be absent from all reptiles, while tail regeneration has, for example, been reported from the Yacare caiman by Dathe [22] and illustrated in a *Caiman crocodilus* by Kälin [20] and is, of course, a well-studied phenomenon in Lacertilia. A recent study by Xu et al. [23] that reviewed the earlier literature on tail regeneration in crocodilians has shown that long and apparently regenerated tails were present in several alligators. The regenerates were found to contain mostly irregular, dense connective tissue around a central rod of cartilage. As the studied specimens were all relatively young individuals, this suggested that they must have become amputated early in their lives and that they had then undergone “regengrow” (defined by Alibardi [24] as a combination involving *regen*-eration and *growth*) to replace their lost tail parts).

Even less information on wound repair and injuries, including those to the tail followed by some form of recovery or restoration, is available in connection with snakes. But there is much anecdotal and also some documented information on wound healing available. After surgically creating 1 cm anteroposteriorly, unsutured linear incisions and one circular wound 6–8 mm in diameter in three garter snakes (*Thamnophis sirtalis perietalis*) and four skin incisions and two excisional wounds 6–8 mm across in a further six snakes, Smith & Barker [25] reported that the sequence of epidermal regeneration was fundamentally similar to that of mammals. Henle & Grimm-Seyfarth [26] cite a report of two snakes with two tails by Redi [27] and refer to a review of axial duplication in snakes by Wallach [28], who stated that 6.2% of 505 examined snakes had tail duplications. However, it remained unclear as to whether the snakes had already possessed the tail duplications during the time they had hatched from the egg or had acquired them later, possibly as a response to an injury.

Whether shorter tails in snakes actually represent a true form of autotomy or are the results of amputations incurred during attacks by other animals, followed by some healing and repair, or whether they had better be referred to as pseudo-autotomies (as suggested in Costa et al. [29]), is a question still not fully resolved. Incidentally, although not snakes but equally limbless reptiles, worm lizards of the family Amphisbaenidae frequently experience tail loss (most likely through injury); yet, regeneration does not occur [30,31]. On the other hand, the occurrence of secondary cartilage in a non-avian dinosaur embryo has been reported by Bailleul et al. [32] and to what extent in reptiles, generally, including Amphisbaenidae, flat bones like those of the skull can be repaired after damage remains to be demonstrated despite an earlier report by Irwin & Ferguson [33], who declared that progenitor cells of reptilian dermal bone “are not capable of forming secondary cartilage”.

### 1.2. Focusing on Tail Autotomies in Reptiles

The processes of autotomy and tail regeneration were likely already present in Captorinomorphs [34] before rhynchocephalians like *Sphenodon* and squamates split apart in the Triassic and these processes were inherited by the two evolutionary lineages of lepidosaurians (Figure 1A) [4]. Since the beginning of their evolution, lizards possessed long tails from which through the phenomenon of autotomy the distal part could be released [34,35]. Based on the evidence of fossil specimens, lizards have always been in an intermediate level of the trophic chain, and were potential prey of both the ruling diurnal archosaurians of the time and nocturnal early mammalian-like reptiles and early mammals [1,2]. Although speculative, it seems likely that without autotomy that facilitated tail loss under predatory or fighting activities, lizards could have faced extinction; instead, however, they became the dominant group of reptiles living today.

In conjunction with autotomy, lizards also evolved a process of regeneration of the tail as a prominent process of post-embryonic development and differentiation. The ability of lizards to regenerate the tail is present to different degrees among extant lizard families [36,37,38], but can vary even within families [39]. Although an enormous amount of scientific literature exists on tail regeneration in lizards, rather little is known in connection with recoveries of other organs and tissues such as skin, scutes, optical nerve, spinal cord, etc. (Figure 1B) [3,40,41,42]. A report by Jacyniak & Vickaryous [43] on cardiomyocyte proliferation in the leopard gecko (*Eublepharis macularius*) showed that cell cycling by cardiomyocytes occurred in this species, but that it was not impacted by caudal autotomy.

Controlled and therefore deliberate tail loss has been reported from a variety of snakes, most notably colubrids, e.g., *Dolichophis caspius* and *Natrix tesselata* by Crnobrnja-Isailoic et al. [44], *Xenochrophis piscator* by Ananjeva & Orlov [45], and *Natriciteres* spp. by Broadley [46]. Caudal autotomy in the eastern garter snake, *Thamnophis s. sirtalis* was reported by Cooper & Alfieri [47]. However, regarding convincing evidence that following autotomy regeneration of lost tails in snakes takes place (if it occurs at all), still needs to be presented. It seems that in snakes replacing autotomized tails by functional regenerates either does not happen at all or is very rare and then restricted to just some families. Only Loveridge [48] and Sharma [49] reported tail autotomies in snakes with subsequent regeneration: the former in connection with members of the genus *Psammophis* spp. (Lamprophiidae) and the latter in connection with *Amphiesma stolatum* (Colubridae). Regarding amphisbaenian worm lizards, a largely fossorial group of limbless squamates, Gans [30] states that they are apparently incapable of regenerating lost tails.

However, in the New Zealand tuatara the phenomenon of replacing a lost tail (or a part of it) does occur. This rhynchocephalian reptile with an ancestry dating back over 220 million years, is a species categorised by the Interational Union for the Conservation of Nature (IUCN) as being of “least concern (https://en.wikipedia.org/wiki/Tuatara accessed 28 August 2021). The tuatara is nowadays occurring only in New Zealand, where it is of cultural significance to the indigenous Maori community as well as to other New Zealanders. The species is adapted to cold-temperate climate conditions [50,51] and as a consequence when compared with squamates from warmer climates, most processes in tuatara require more time. Although tuatara can regenerate variable portions of its tail, unsurprisingly the regeneration is very slow and because the latter happens contemporaneously with body growth, it can be considered to represent regengrow [24,52]. Since the last comprehensive review on lizard caudal autotomy in which tuatara are mentioned is that of Batemen & Fleming [53] more than ten years ago, it seemed pertinent to produce an update, specifically focusing on tuatara on account of its pivotal phylogenetic position in the Rhynchocephalia as a sister-group of the squamates.

## 2. Caudal Autotomy in Tuatara

### 2.1. Sample Acquisition and Methodology

Our review and all micrographs are based on the 1988 and 1989 material, which was obtained with the assistance from the Department of Conservation through a permit issued by Mr. Ian Govey and from Victoria University’s tuatara culture, headed by Dr. Mike Thompson. New material for the images in this review were not used and none of the images, obtained through standard methods explained in detail in all of our earlier publications, had been published before. There are therefore no copyright issues.

To study how tail autotomy in the tuatara affected the histological and cellular responses in the regenerate, observations by light and electron microscopy were carried out and combined with cytochemical analyses. After fixation in 4% or 10% buffered formaldehyde, regenerating blastema or 2–4 mm long pieces of the regenerating tail were embedded in wax, Epon, or LR-white resins for sectioning, using a microtome (for wax) or an ultramicrotome (for Epon and resins). Sections were stained using Haematoxylin-Eosin, 1% Toluidine blue, or Palmgren silver stain for nervous and connective fibres. Tissue fixation, embedding, staining, and sectioning techniques have been basically the same in all of our previous investigations involving tuatara as well as other reptiles and detailed descriptions of the histological and ultrastructural preparation methods are given in [42,52].

### 2.2. Tail Regeneration in the Tuatara Represents a Case of Regengrow

**Figure 2:**
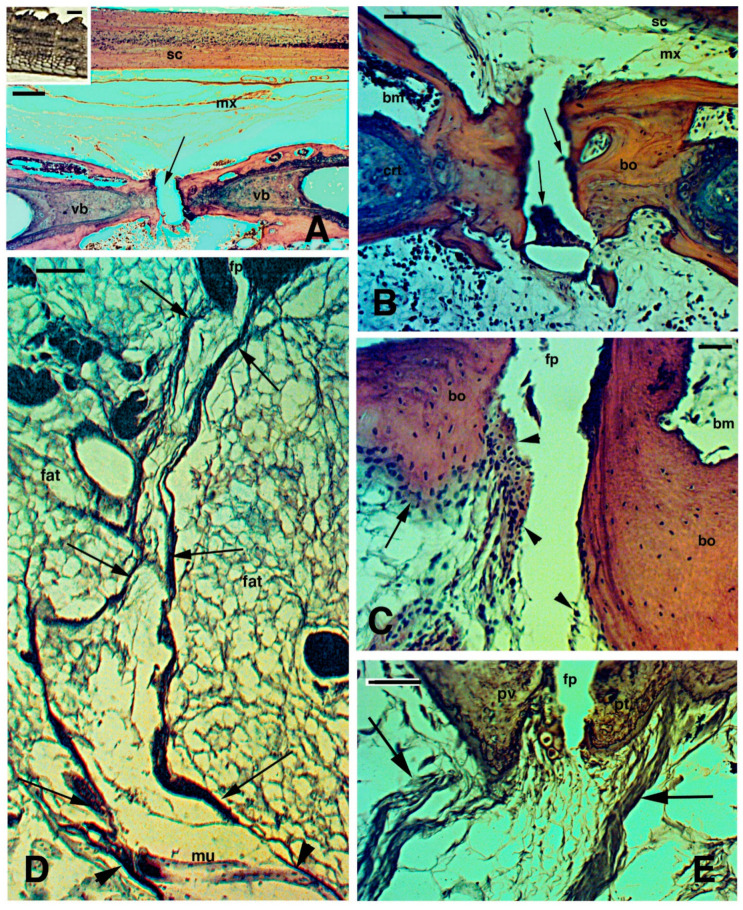
Histological images (stained with Haematoxylin-Eosin in (**A**–**C**), and Palmgreen stain in (**D**,**E**)) of the caudal vertebrae of a normal tail in the tuatara (inset in Figure **A**, Bar, 2 mm). (**A**), image showing the spinal cord, meninx (artifactually dislocated after sectioning), and ventrally the vertebral body with the intra-vertebral fracture plane indicated (arrow), which has artifactually been separated during sectioning. Bar, 100 μm. (**B**), closer view of fracture plane with fragments of blue-stained cartilaginous cells (arrows), probably dislocated from the articular surfaces during sectioning. Bar, 100 μm. (**C**), close-up of the intra-vertebral splitting plane where numerous fibro-cartilaginous/connective cells (arrowheads) are present in continuation with the periosteum (arrow). Bar, 50 μm. (**D**), peri-vertebral region rich in fat cells and loose connective showing two fibrous bundles (arrows) connecting pre- and post-vertebral bodies at the fracture plane to the inter-muscle connective septa (arrowheads). The image represents the plane of autotomy of the tail, which continues (not shown) into the dermis to externally reach the scales. Bar, 100 μm. (**E**), detail of the fibrous bundles (arrows) connected to the pre-vertebral and post-vertebral bodies at the fracture or splitting autotomous plane. Bar, 50 μm. **Legends**: bm, bone marrow; bo, vertebral bone; crt, cartilage; fp, fracture plane; mx, meninx; pt, post-vertebral body (more caudal) at the fracture plane; pv, pre-vertebral body (more rostral) at the fracture plane; sc, spinal cord; vb, vertebral body. **Note**: All micrographs are based on material obtained in 1988 and 1989 through a permit issued by Mr Ian Govey of the New Zealand Department of Conservation and Dr Mike Thompson of Victoria University, Wellington (New Zealand). The material was used in Alibardi and Meyer-Rochow [52,55] and all subsequent publications on tuatara by these authors.

Like numerous species of lizard, the tuatara possesses autonomous fracture planes in the tail [35,54,55]. The histological analysis of autotomous tail vertebrae of *Sphenodon*, designated as pygous vertebrae by Seligmann et al. [56], showed that the splitting or fracture plane contains small cells resembling blood elements of the bone marrow and additional flat perichondrial cells or even chondrocytes/chondroblasts in continuity with the fibro-cartilaginous tissue of the vertebral bone at the splitting surface (Figure 2A–C). The fibro-cartilage is contacted at the fracture plane by numerous connective fibrils in separated pre-fracture and post-fracture vertebral bodies. The fibrils give rise to 15–30 µm thick fibrous bundles crossing the peri-vertebral adipose tissue and are in continuation with the inter-muscle septa (Figure 2D,E). The latter terminate in the dermis and contact the basement lamella of the scales, thereby forming the autotomous planes of the tuatara tail [55], as observed also in lizards [35,57].

The microscopic observation of the cells present in the fracture plane of the vertebrae indicates that they resemble those described for lizards [35,57], which demonstrates the presence of stem/pro-cartilaginous cells in this region where also 5BrdU-Long Retaining Labelled cells (LRC) have been observed [58]. The presence of putative stem elements that can give rise to new cartilaginous cells explains the production of a large cartilaginous tube after the tail is autotomized along the fracture (autotomous) plane. The presence of remaining cartilaginous/chondroblast cells within the caudal vertebrae of *Sphenodon*, suggests that cartilage cells for tail regeneration could also derive from the inter-vertebral region when the amputation occurs at this level or after vertebral ablation [56].

**Figure 3:**
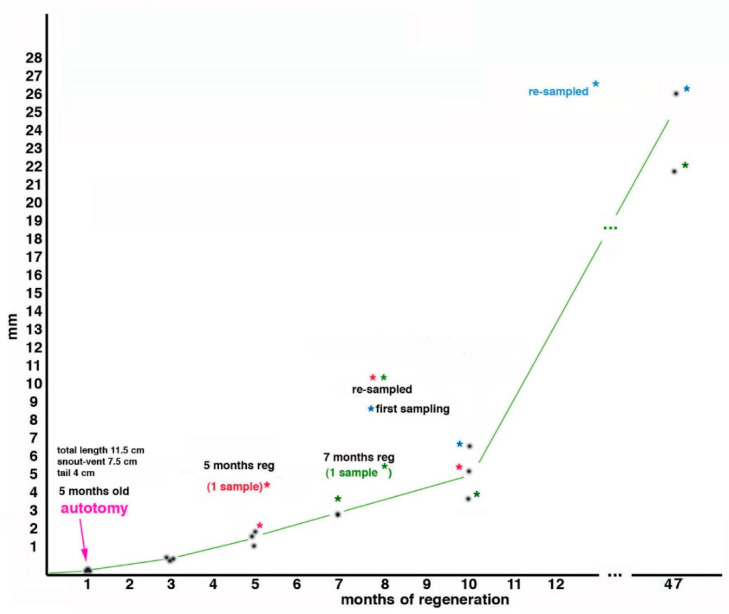
Growth of the regenerating tail during 47 months associated with body regeneration in three tuatara (indicated by asterisk of different coloration), received initially at 3 months of age, autotomized at 5 months of age at about half-distal length of the tail, measured and sampled at different periods thereafter (see text for further explanations). The ordinate refers to the growth of the regenerate, while the abscissa refers to time.

In the study by Alibardi and Meyer-Rochow [52], during the first 4 years and 5 months of life (47 months in regeneration + 5 months since they hatched = 53 months of life in total), three young tuatara grew about 25% (snout-vent length, from 7.5 cm to 10.6 cm) during which time they were autotomized two times to study the regeneration of their tails (Figure 3). The first tail autotomy was performed on number 1 specimen at about 5 months after birth, and the sample was collected at 5 months of regeneration (=10 months of age); a second sample came from another individual at 7 months of regeneration (=12 months of age), and 3 samples represented 10 months of regeneration (2 re-amputated and one at the first amputation, but all at an age of the tuatara of 15 months). After 37 months from the last amputation, the average body lengths in 2 juveniles were15.5 cm (with an expected snout-vent length of 16.3 cm, the individuals were therefore 0.8 cm shorter than had been expected). This value corresponded to a loss of 5% total body length but to 24.6% of tail growth due to the 2 repetitive regenerations (Figure 3, the graph). Because of the long duration of tail regeneration in the tuatara, it is likely that in addition to the initial wound healing, blastema formation, and the differentiation of small muscle segments and an axial cartilage, the slow process of growth contributed to the apparent regeneration of the tail [52,59,60]. Tail re-regeneration was also studied in the skink *Egernia kingii* by Barr et al. [60]: it was present in 17.2% across three populations and the authors concluded that “the ability to re-regenerate may minimise the costs to an individual’s fitness associated with tail loss, efficiently restoring ecological functions of the tail”. Although slower in the tuatara [61] than in the aforementioned skink, re-regeneration of the tail in tuatara may provide similar benefits.

If we now compare this finding with a previous study on regenerated tails in *Sphenodon*, in which it was calculated that juveniles grow at about 1.14 cm/year [55], we have to note that in our study we observed a much lower value, namely 0.79 cm/year, based on snout-vent length, which increased by 3.1 cm in 47 months (3.92 years). This corresponded to an about 31% lost growth by comparison to 1.14 cm/year, and is therefore much higher than our calculated 5% and 24.6% figures. Despite the different absolute percentages of body length loss in the two examples, it appears that repeated tail regeneration did influence normal growth in *Sphenodon*. Since the bodies of these juveniles grew about 25% in length in 47 months (about 4 years), this suggests that the tissues observed in the regenerated tails must have also grown at a similar rate.

The influence of somatic growth over the years affects all tissues including those in the regenerating tail [61] and especially muscles and axial cartilage, a process that is indicative of regengrow (defined earlier in this paper and in Alibardi [24]). Although small muscles are regenerated during the first year following amputation [52], large muscle masses are observed in the longer regenerated tails of mature individuals [55, and present study], and they are equally regenerated by juvenile stages during the years following tail loss. These large masses of muscle and cartilaginous tissues have also been observed in two adult individuals that we have analysed, a male and a female, which presented long-term regenerated tails of 2.8 and 10.4 cm. An older juvenile with a regenerating tail of 1.7 cm, produced from a previous regenerated tail, also showed a complex tissue organization in the new tail [51,59,62]: see next paragraph.

**Figure 4:**
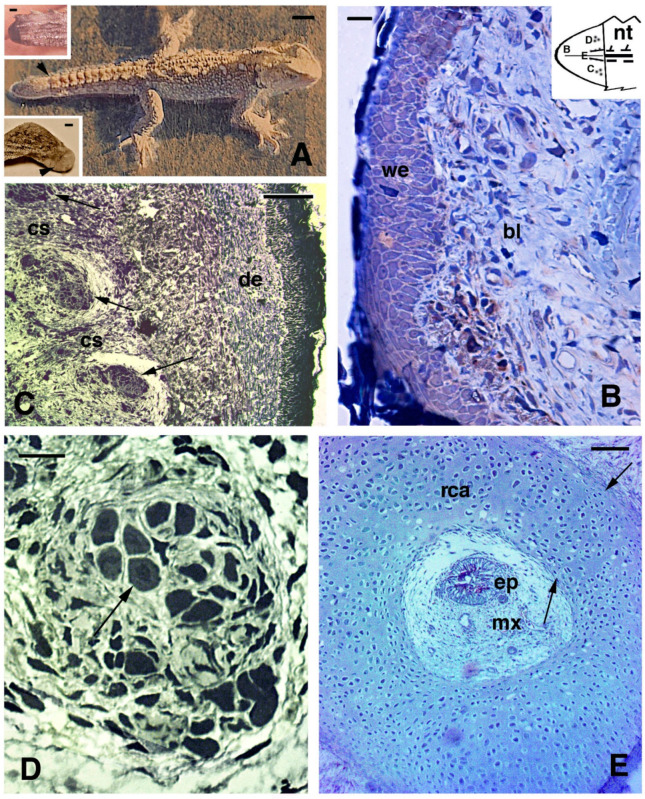
Gross aspect (**A**) and histology of regenerating tail ((**B**–**E**), Toluidine blue stain). (**A**), juvenile of about 4 years of age with regenerated tail (arrowhead). Bar, 1 cm. In the upper inset (Bar, 1 mm) a blastema of about 2 months is shown. The scar (arrowhead) depicted in (A) lower inset formed after about 3 months following limb amputation. (**B**), blastema at about 3 months with a loose connective covered by a thick wound epidermis. Bar, 10 μm. This schematic inset shows a blastema with the regions shown in (**B**–**E**). (**C**), proximal area of cross-sectioned conical blastema of 10 months post-autotomy showing three pro-muscle aggregates (arrows) separated by forming connective septa. A dense dermis is present beneath the cornified epidermis. Bar, 50 μm. (**D**), detail of a muscle bundle at 10 months post-autotomy. The arrow indicates a myotube in cross-section. Bar, 10 μm. (**E**), cross sectioned central cartilaginous cylinder surrounding the ependymal canal in proximal regions of a cone of 7 months post-autotomy. Arrows point to the outer and inner perichondria. Bar, 50 μm. **Legends**: bl, blastema; cs, connective septa (forming intermuscle); de, dermis; ep, ependymal canal; mx, meninge; nt, normal tail (stump containing vertebrae and spinal cord); rca, regenerated cartilage; we, wound (regenerating) epidermis. **Note**: All micrographs are based on material obtained in 1988 and 1989 through a permit issued by Mr Ian Govey of the New Zealand Department of Conservation and Dr Mike Thompson of Victoria University, Wellington (New Zealand). The material was used in Alibardi and Meyer-Rochow [52,59,60,62].

Wounds to the limb, like in lizards may heal, but as with lizards, they do not lead to a regenerate in *Sphenodon*. This was also observed after accidental injury where a juvenile lost the anterior left foot but, after 3–15 months only developed a pale and scaled-over scar covered by very small scales (lower inset in Figure 4A). Although digits also do not regenerate [56], if a toe clip (a method for identifying individuals that is now generally avoided) is performed incorrectly only just beneath the base of the claw, then the tissue and claw do sometimes regenerate with a stub claw (A. Cree, personal communication, 2021). Other cases of wound healing are not known or have not been described microscopically in *Sphenodon* and New Zealand veterinarians, who from time to time have to treat tuatara, have a saying that “tuatara get sick and recover on tuatara time”, meaning that recovery is very, very slow (M. Jolly, personal communication 2021).

The question, of course, arises what the benefits to tuatara could have been to evolve and maintain the ability to autotomize its tail and grow a new one, even if the regrowth takes a very long time [60]. The high frequency of tail regeneration, estimated by one of us (LA) on the basis of field observations on Stephens Island to be around 80%, suggests that the loss of the tail is a common event in this species. These days tail loss is probably primarily due to predation attempts of adult individuals on juveniles, attacks by large centipedes such as *Cormocephalus rubriceps* on very small tuatara, and fighting during courtship among adults [61]. Predation by flighted predators such as harriers and kingfishers is also known and until its extinction in 1914, predation by the Laughing Owl *Ninox (Sceloglaux) albifacies* most likely occurred as well.

But in the past—for at least 20 million years—tuatara had to cope with more formidable predators, among them being large flightless birds such as *Aptornis otidiformis* in the North Island and *A. defossor* in the South Island known as adzebills [63]. These extinct predators appear to have been by and large diurnally active [63] and that may have been a factor why tuatara, despite possessing an eye dominated by photoreceptive cones characteristic of diurnal species Meyer-Rochow et al. [64], became a largely nocturnal predator with vision adapted to very low light intensities [65,66]. It is obvious that the nocturnal lifestyle, however, did not eliminate the need for tail autotomy, but whether regeneration has always been as slow as it is now or was faster in the past is difficult to ascertain.

### 2.3. Histology of Regenerating and Regenerated Tails in the Tuatara

A summary of our earlier studies is presented in this paper, but more detailed information on specific aspects of the processes involved in regenerating different tissues is available from our previously published and more detailed analyses [51,58,59,62,67,68,69,70]. After tail autotomy or amputation the stump heals very slowly and to complete re-epithelialization (at 23–24 °C) about 1 month is required. A regenerative blastema is visible from about 2–3 months (Figure 4A, upper inset), but in the following months from then on the stump grows considerably more slowly (Figure 3). At about 3 years (37 months) from the second amputation, the tail has grown, but is much shorter than the original, often with a club-like shape (Figure 4A). An amputated limb forms a scar after 3 months from the amputation, but it does not grow into a limb even after 10–15 months, and does not regenerate any further (Figure 4, lower inset).

Microscopic investigations show that the wound (regenerating) epidermis covering the blastema of 1–2 mm is multilayered and forms an initially soft corneous layer, while underneath loose connective tissue is present containing mainly fibroblasts, blood vessels, sparse nerves, and blood cells (Figure 4B). In regenerating cones of 3–6.5 mm at 5–10 months also some pro-muscle aggregates are recognizable in the more proximal regions close to the original tail. Here they form, just like in lizards, 12–16 very small muscle groups identifiable in cross section, each one made up of a limited number of myotubes (15–30; Figure 4C,D). Most of the regenerating cones at 10–15 months post-amputation are composed of connective tissue containing fibrocytes and numerous collagen fibrils with irregular orientation. In the central part of the cones of 3–6.5 mm in length (Figure 3), a cartilaginous cylinder is formed that shows flat chondroblasts at the external and internal periphery, as observed in longitudinal and cross-sections (Figure 4E).

**Figure 5:**
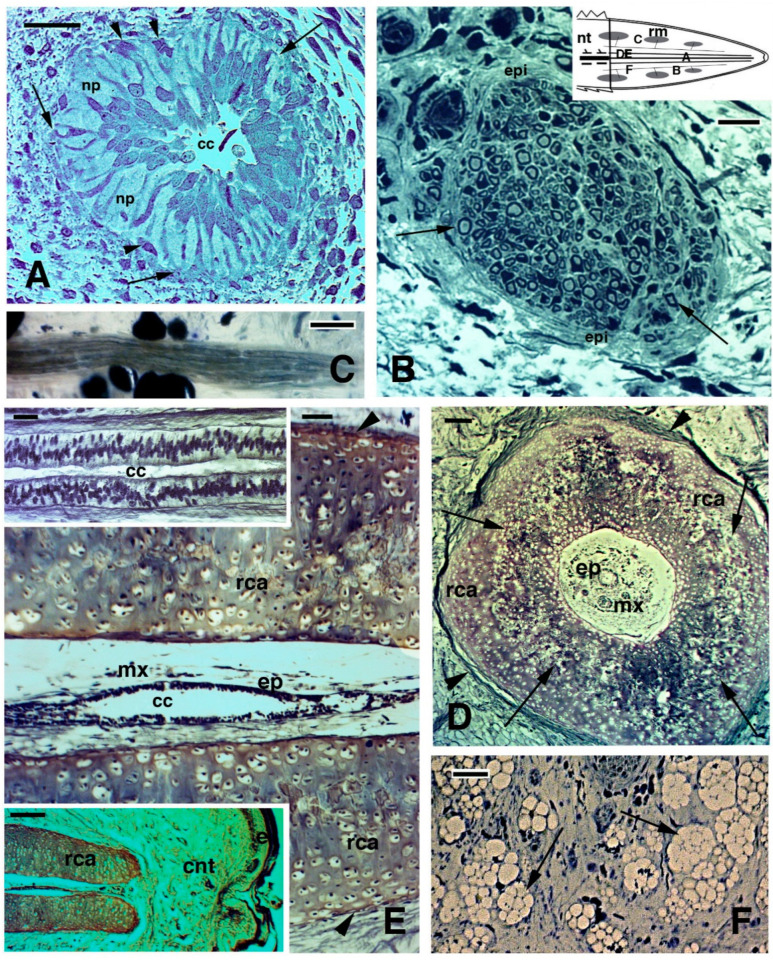
Histology of regenerating tail (**A**,**B**,**F**) Toludine blue stain; (**D**,**E**) Palmgreen stain). (**A**) cross-sectioned ependymal tube showing the elongation of ependymal tanicytes ending on the basement membrane (arrows). Arrowheads point to glial cells detached from the ependymal epithelium. Bar, 10 μm. (**B**), cross-sectioned myelinated (arrows) nerve at 10 months regeneration. Bar, 10 μm. The schematic drawing shows the indicated positions of the figures. (**C**), longitudinal section of myelinated nerve 10 months post-autotomy. Bar, 10 μm. (**D**), cross section of the cartilaginous tube in a long regenerated tail of unknown age. Arrows indicate intra-cartilaginous areas of calcification/degeneration. Arrowheads indicate the fibrous connective contacting the perichondrium. Bar, 50 μm. (**E**) longitudinal section of axial cartilage in an old regenerate of unknown age. The outer perichondrium is indicated by arrowheads. Bar, 20 μm. The upper inset (Bar, 10 μm) details the pseudostratified ependymal epithelium. The lower inset (Bar, 50 μm) instead shows the apical end of the cartilaginous tube, close to the connective tissue of the tip of the regenerated tail. (**F**), numerous fat cells (arrows) are present around the cartilaginous tube. Bar, 20 μm. **Legends**: ca, regenerated cartilage; cc, central canal; cnt, connective (fibrous) tissue; e, epidermis of neogenic apical scale; ep, ependymal epithelium; epi, epinevrio; mx, meninx; np, areas occupied from axons and neuropile; nt, normal tail (stump);rca, regenerated cartilage; rm, regenerating muscles/myomeres. **Note**: All micrographs are based on material obtained in 1988 and 1989 through a permit issued by Mr Ian Govey of the New Zealand Department of Conservation and Dr Mike Thompson of Victoria University, Wellington (New Zealand). The material was used in Alibardi and Meyer-Rochow [52,58,59,62].

Inside the cartilaginous tube a loose meninx with numerous blood vessels and a simple spinal cord are regenerated (Figure 4E and Figure 5A). At 5 months of regeneration the spinal cord is formed by ependymal cells, most of which appear as elongated tanicytes terminating into the external basal lamina. The pale spaces among tanicytes are occupied with axons and neuropilar elongations, while rare glial and neuronal cells are present. Among the fibrous connective tissue located outside the cartilaginous cylinder, various amyelinic and myelinated nerves are present, and their terminations reach the apex of the regenerating tail at 5–10 months of tail regeneration (Figure 5B,C). Numerous fat cells are formed in the proximal regions of regenerating 3–6.5 mm large cones.

After 37 months from the last collection (the last sampling done on 2 out of the initial 3 specimens), the regenerated tail appeared to consist mainly of irregular dense connective tissue, with large accumulations of fat cells around the central cartilaginous tube where calcification is predominantly noticeable among internal isogenic groups (Figure 5D–F). Flat chondroblasts likely forming a perichondrium are seen in the inner and outer periphery of the cartilage, while only tanicytes with a pseudostratified organization are present in the regenerated spinal cord. Segmental muscles remain limited in these regenerated tail of about 3 years, but the 2–3 amputations carried on during this period (Figure 3) may have stimulated excessive fibrosis. This is also indicated by the histological analyses of regenerated tails from older specimens of unknown age [52].

**Figure 6:**
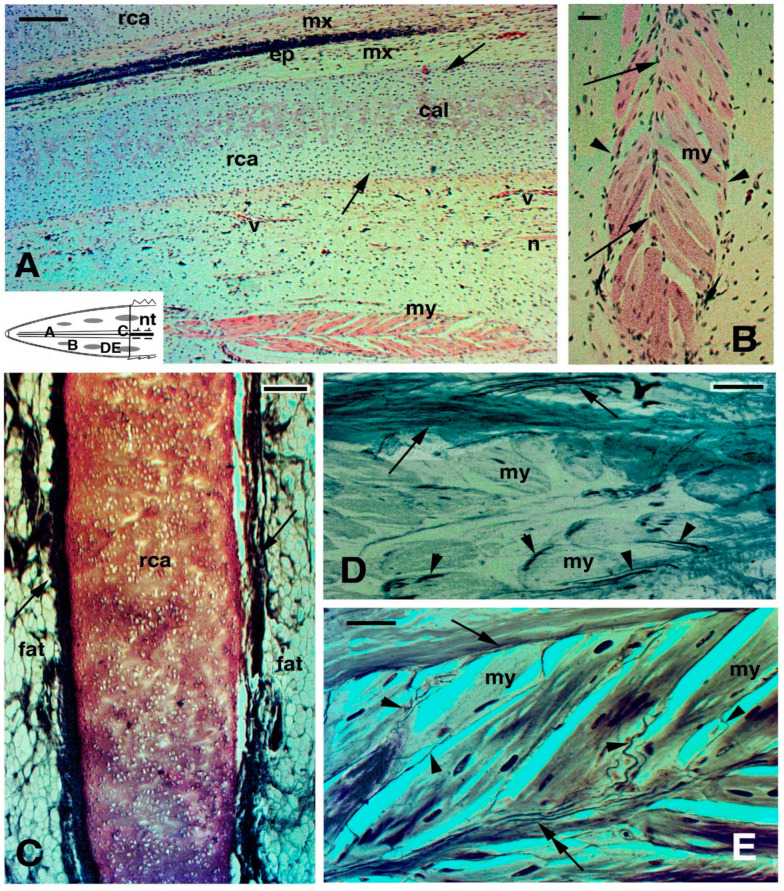
Histology of regenerating tail of unknown age (**A**,**B**) Haematoxylin-eosin stain; (**C**–**E**), Palmgreen stain). (**A**), mid-apical region showing a distinctly cellular cartilage with outer and inner peripheries (arrows) representing the perichondrium. Bar, 50 μm. The schematic drawing shows the indicative positions of the following figures. (**B**), detail on a forming, leaf-like myomere, with an axial intermuscle connective (arrows) and external limiting connective septa (arrowheads). Bar, 10 μm.(**C**), tangential longitudinal section of the axial cartilage surrounded by fat connective tissue in an old (age unknown) regenerating tail. Arrows indicate the fibrous layer in contact with the perichondrium. Bar, 50 μm. (**D**), detail showing nerve fibres coursing within the outer connective septum (arrows) and other nerves entering the myofibres (arrowheads). Bar, 10 μm. (**E**), additional details of nerve endings from peripheral (arrow) and central myoseptum (double arrow) entering myofibres (arrowheads). Bar, 10 μm. **Legends**: cal, beginning of cartilage calcification; ep, ependyma; fat, connective tissues rich in fat cells; mx, meninx; my, multinuclear myotubes/myofibres; rca, regenerated cartilaginous tube; v, blood vessels. **Note**: All micrographs are based on material obtained in 1988 and 1989 through a permit issued by Mr Ian Govey of the New Zealand Department of Conservation and Dr Mike Thompson of Victoria University, Wellington (New Zealand). The material was used in Alibardi and Meyer-Rochow [52,58,62].

In a smaller individual, a regenerating tail of 1.7 cm shows small muscle aggregates formed by multinucleated myotubes that exhibit a “leaf-like shape” in the flat plane of the section, as myotubes are attached with a central connective myoseptum and 2 external myosepta (Figure 6A,B). Very large myotomes are instead observed in the other two large specimens of unknow age, possessing long regenerated tails (2.8 and 10.4 cm), as has also been illustrated in other cases (see Figures 10 and 11 in: [55]). Old regenerate/regengrow tails also contain large deposits of peri-cartilaginous fat, and more externally substantial muscle bundles with a high innervation score, as observed using the Palmgreen silver staining method for nerve fibres. The latter staining evidences an external innervation of myotubes, starting from nerve fibres crossing the external and internal connective septa and penetrating inside the myotubes or the muscle fibres in more proximal myomeres of the regenerated/regengrow tail (Figure 6D,E).

**Figure 7:**
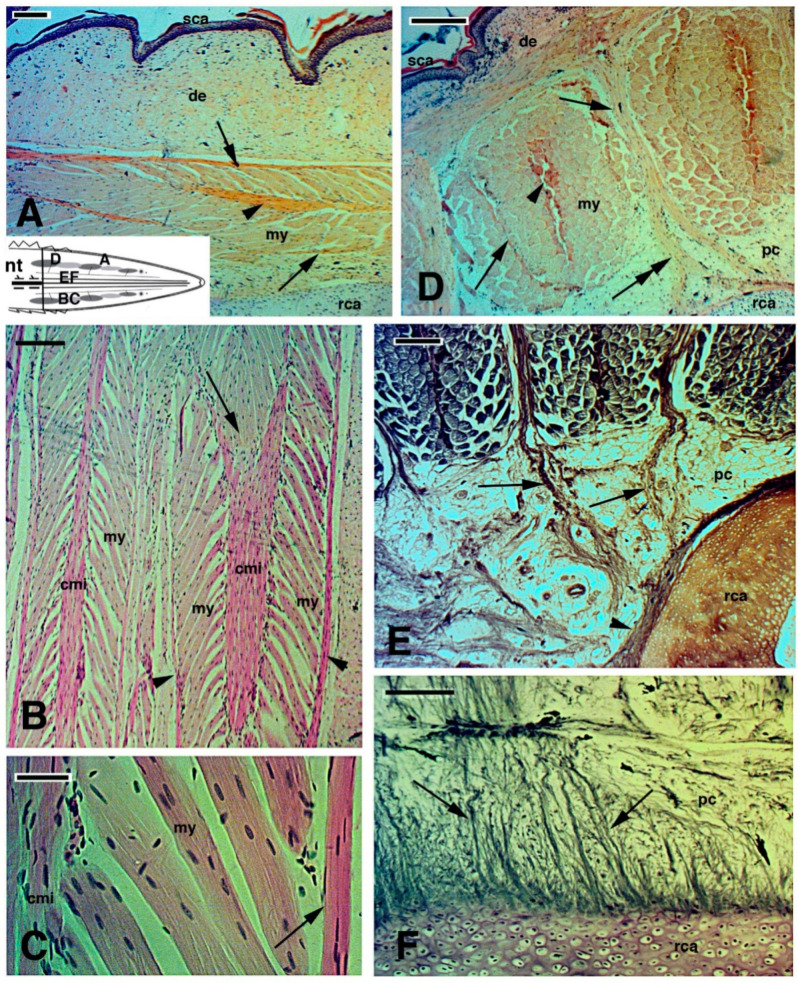
Images derived from different areas of old regenerated tails of unknown age (**A**–**D**), Haematoxylin-Eosin stain; (**E**,**F**), Palmgren stain). (**A**), detail of regenerated muscles with outer myoseptum (arrows) and inner myoseptum (arrowhead). Bar, 50 μm. The schematic drawing shows the positions of the following figures. (**B**), detail to show the leaf-like organization of regenerated muscles. The arrow shows a muscle cone of a myomer that is inserted in the following myomer through the central myoseptum. Bar, 50 μm. (**C**), detail of multinucleated muscle fibres attached to the central and lateral (arrow) myosepta. Bar, 10 μm. (**D**), cross sectioned muscle bundles close to the tail stump showing the central myoseptum (arrowhead), the lateral myosepta (arrows) that are in continuation (double arrow) with a fibrous bundle connected to the central cartilaginous tube. Bar, 50 μm. (**E**), other details showing more clearly the fibrous connections (arrows) between intermuscle connective tissue and the circular fibrous tissues (arrowhead) contacting the cartilaginous tube. Bar, 50 μm. (**F**), longitudinal section showing numerous fibrous bundles (arrows) connecting the cartilaginous tube with surrounding tissues, including muscles (here not included/visible). Bar, 50 μm. **Legends**: cmi, central connective myoseptum; de, dermis; my, myofibres; nt, normal tail (stump); pc, pericartilaginous connective tissue; rca, regenerating cartilage; sca, scales (neogenic). **Note**: All micrographs are based on material obtained in 1988 and 1989 through a permit issued by Mr Ian Govey of the New Zealand Department of Conservation and Dr Mike Thompson of Victoria University, Wellington (New Zealand). The material was used in Alibardi and Meyer-Rochow [51,58].

Large segmental myomeres occupy extensive areas of regenerated tail at 2.5–10 cm from the tip of the new tail. They give rise to over 20 muscle bundles in cross section whose dimensions decrease from proximal regions toward the apex (Figure 7A, inset). In his study, Ali [55] observed about 40 bundles of “regenerated” muscles, which did, however, exhibit smaller dimension than the original intrinsic and extrinsic tail muscles. As indicated above, it is likely that these large segmental muscles derive from a long process of growth superimposed on the initial regeneration of pro-muscle aggregates, observed in the early regenerating tail. The structure of the large myomeres maintains the original leaf-like shape in a flat plane, with a thick, central connective septum and two peripheral septa to which the muscle fibres are attached (Figure 7A–C). Although the precise three-dimensional shape of these segmental muscles remains un-determined, it appears that the terminal cones of one muscle segment insert into at least one, perhaps even two, successive myomeres, forming an acute zig-zag conformation (Figure 7A,B,D,E; see also: [55]). In both longitudinal and cross sections of the regenerated tail, numerous variably thick inter-muscle connective septa contact the fibrous periosteum of the cartilaginous tube (Figure 7E,F). This anatomical connection or arrangement suggests that muscle and axial skeleton are mechanically integrated, and even that muscle contraction (after nerve impulse registration in the regenerated/grown muscles) is transmitted to the axial skeleton, although the tail in tuatara appears quite stiff and little capable of bending.

## 3. Immunohistochemical Considerations

### Cell Proliferation in Old Regenerated Tails Supports the Concept of Regengrow

**Figure 8:**
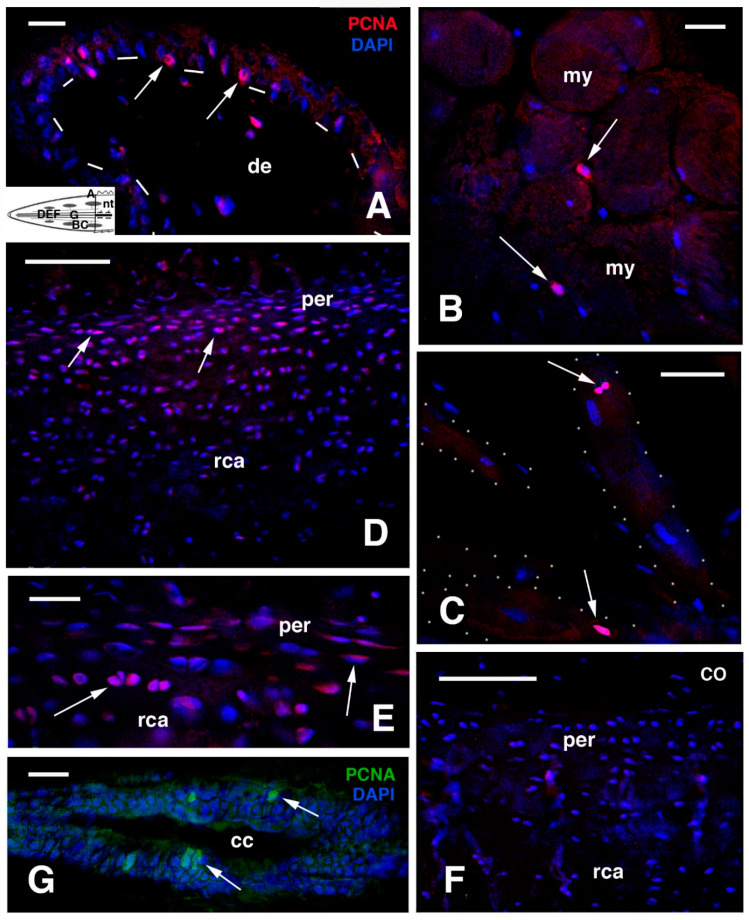
Immunofluorescence using the PCNA antibody. (**A**), neogenic scale located near the tail stump with sparse labelled cells (arrows). Bar, 10 μm. The schematic inset shows the position of the following images. (**B**), Cross-sectioned proximal muscles. Arrows show two labelled cells. Bar, 10 μm. (**C**), obliquely-sectioned muscle fibres (outlined by dots) with labelled nuclei (arrows). Bar, 10 μm. (**D**), external part of the cartilaginous cylinder with labelled flat chondroblasts (arrows) especially abundant in the perichondrium. Bar, 50 μm. (**E**), detail of labelled cells (arrows) in the perichondrium. Bar, 20 μm. (**F**), immunonegative control sections (CO) of cartilaginous cylinder. Bar, 50 μm. (**G**), detail on the ependyma of medio-proximal region showing few labelled nuclei (arrows) Bar, 10 μm. **Legends**: cc, central canal; de, dermis; my, myofibres/myotubes; nt, normal tail (stump); per, perichondrium; rca, regenerated cartilage. **Note**: All micrographs are based on material obtained in 1988 and 1989 through a permit issued by Mr Ian Govey of the New Zealand Department of Conservation and Dr Mike Thompson of Victoria University, Wellington (New Zealand). The material was used in Alibardi and Meyer-Rochow [67].

**Figure 9:**
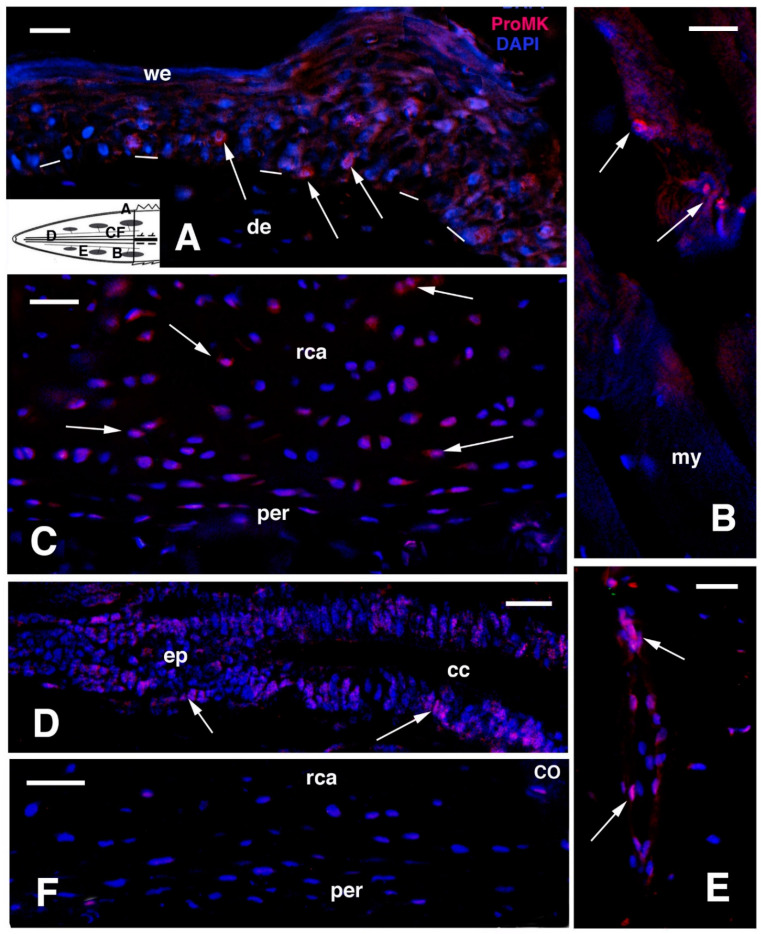
Immunostaining using the proliferation marker antibody (KI-67). (**A**), epidermis (dashes underline the basal layer) of proximal scale (arrows point some labelled nuclei). Bar, 10 μm. The schematic inset shows the position of the following images. (**B**), proximal muscles with few labelled nuclei (arrows). Bar, 10 μm. (**C**), external part of the cartilaginous cylinder with few labelled cells (arrows) in the perichondrion and inside isogenic groups. Bar, 20 μm. (**D**), medium-apical ependyma with few labelled nuclei (arrows). Bar, 10 μm. (**E**), small blood capillary with various labelled endothelial cells (arrows). Bar, 10 μm. (**F**), immunonegative control section of external region of the cartilage and perichondrion. **Legends**: cc, central canal; de, dermis; ep, ependymal epithelium; my, myofibre; nt, normal tail (stump); per, perichondrium; rca, regenerated cartilage; we, wound epidermis (corneous layer). **Note**: All micrographs are based on material obtained in 1988 and 1989 through a permit issued by Mr Ian Govey of the New Zealand Department of Conservation and Dr Mike Thompson of Victoria University, Wellington (New Zealand). The material was used in Alibardi and Meyer-Rochow [67].

In order to determine whether cell proliferation is active and regengrow continues in old regenerated tails, we have studied the main sites of proliferation in the three old regenerated tails found in nature and available to us [52,58,59,62] (Figure 8 and Figure 9). From other sections obtained from the previously utilized material, a method to retrieve immunoreactivity has been used on wax-embedded tissues in order to detect PCNA (Proliferation Cell Nuclear Antigen) labelled nuclei. Longitudinal sections of regenerated tails were de-waxed, rinsed in water, immersed in 0.1 M citrate buffer at pH 5.6, and then treated in a microwave oven for 5–6 min for antigen retrieval. Following that procedure, the sections were incubated with a mouse antibody against PCNA (Sigma, Burlington, MA, USA), rinsed and immunolabelled with anti mouse-TRITC (red) or-FITC (green), counterstained for nuclei using the blue fluorescent DAPI, and observed under a fluorescent microscope.

PCNA-labelled nuclei were few in number and mainly observed in the epidermis and at the periphery of the axial cartilaginous tube; they were only occasionally detected in other tissues (Figure 8). In the regenerated scales, few labelled nuclei were visible in the basal layer of the outer scale surface (dorsal longer side of scales), and sparse and infrequently labelled nuclei were seen associated with the surface of segmental muscles or even inside the long regenerated muscle fibres (Figure 8A–C). Numerous PCNA-labelled cells were instead present in the external and internal surfaces along the cartilaginous tube, where smaller and flat chondroblasts were present. Labelling, however, became less intense and disappeared altogether in the central part of the cartilage (Figure 8D,E).

Control sections did not show labelled cells (Figure 8F). Few PCNA-labelled nuclei were also observed in the apical regenerating ependymal tube, localized inside the cartilaginous cylinder (Figure 8G). Labelled nuclei were rarely observed in the connective and adipose tissues present around the cartilaginous cylinder, in blood vessels and nerves. Therefore, the two main sites of proliferation, i.e., the external regions of the cartilaginous tube and scales, indicate that growth is very low but still active in some of the tissue of the old regenerating tails, a process likely also present in the skeletal-muscle apparatus of the remaining body, thus sustaining the idea that a process of regengrow is involved in the tail regeneration of tuatara juveniles and young individuals [52] as it is in other reptiles with continuous growth, Xu et al. [23,24].

The PCNA observations were supported by the use of another cell proliferation marker antibody (KI67-3E6, from Hybridoma Bank, Iowa City, IA, USA). After antigen retrieval, the sections revealed sparse labelled cells (nuclei) in the epidermis of neogenic scales (Figure 9A), and occasionally labelled nuclei within regenerated myofibres (Figure 9B). More frequent labelling was observed in the cartilaginous cylinder, especially in the un-calcified external and internal (perichondrial) regions (Figure 9C). Furthermore, the apical ependymal tube and ampulla showed small numbers of labelled nuclei (Figure 9D). Sparse nerves and various blood vessels and capillaries also contained labelled cells in the endothelium (Figure 9E), but control sections showed no labelled cells (Figure 9F). Therefore it appears that the cartilage maintains the highest proliferation in the regenerated tail, followed by the epidermis (this varies according to the period within the shedding cycle), ependymal canal, blood vessels, and muscles, indicating that these tissues undergo slow growth.

An observation well worth investigating in the tuatara is whether epithelial proliferation near the onset of ecdysis might not contribute to an accelerated production of cells associated with the wound left by the autotomized tail. A scenario such as that has been reported by Smith & Barker [25], who state that “in the few snakes in which the proliferative stage of ecdysis occurred between wounding and necroscopy, this acceleration seemed to occur.” These authors speculate that hormonal manipulation to induce ecdysis could possibly be a means to promote a faster wound repair in squamates. To test this hypothesis in the slowly growing tuatara would be quite a challenge.

## 4. Conclusions and Outlook

In conclusion, the formation and acquisition of the complex anatomy of the regenerated tail in *S. punctatus*, although simpler in view of its ramification and functional range by comparison with the original tail, not only requires a long time; it also lags behind in comparison with the normal growth of this reptile when not yet fully grown to adult size. This process is herewith indicative of regengrow [24,52]. The initial regeneration and tissue organization of the blastema [71], associated with the tuatara’s continuous growth during its lifetime, a feature of its longevity actually doubted by Dawbin [72], determines the size increases of numerous tissues in the new tail. It can lead to hypertrophy in muscles and growth for the continuous appositional addition of chondroblasts for lengthening the axial cartilage and for the replacement of cartilaginous cells that degenerate during calcification in the central region of the cartilaginous tube [52].

The new tail appears to function mainly as a fat repository and as a mechanically functional appendage for balanced locomotion. The tail is also likely to be important for behavioural displays and aggressive encounters, especially in males [61,67]. As among lizards, wound healing and tissue growth in the tuatara provide us with important information on the generally limited capacity for regeneration in amniotes, this information also addresses future interventions aiming to increase healing processes and to decrease scarring in amniotes. This includes humans, and a better understanding of the role that the integument plays as a microbial barrier and protector against injury can result from a more detailed understanding of what happens to the regenerating integument in the tuatara as an extant representative of the earliest amniotes [73,74].

The study of regenerative events can also help to determine evolutionary scenarios. It was, for example, shown that dorsal crest scales and those of the tail spines of the tuatara represent different specializations and have different roles to play [67]. Tail ridge scales and those of the crest spines possess the typical epidermal organization of lepidosaurians with a complete beta-layer, mesos region, and alpha-layer to which in agreement with [75,76,77,78,79] and Alibardi & Meyer-Rochow [42] new keratin and corneous proteins are added and incorporated. This condition corresponds to a post-shedding epidermal phase, i.e., the most common stage found when sampling scales at random in both tuatara and lizards. Modern birds, too, possess scales on their legs, but a molecular and cellular comparison of the chicken’s scutate scales with alligator scales by Wu et al. [79] led these authors to conclude that avian scales and reptilian scales may use different molecular circuits to regulate development and morphogenesis. Besides the processes in the skin, the different calcification pattern of the regenerated axial cartilage between tuatara and lizards [77,80] also remains largely unexplained, but the formation of new cartilage in the outer and inner parts of the axial cartilaginous tube (Figure 5D,E, Figure 8D,E and Figure 9C) further indicates that a process of regengrow is active in *S. punctaus*.

## Data Availability

Data supporting the reported results, including links to publicly archived datasets analyzed or generated during the study, are available on request from LA (Comparative Histolab: lorenzo.alibardi@unibo.it).
